# Creatine supplementation and oxidative stress in rat liver

**DOI:** 10.1186/1550-2783-10-54

**Published:** 2013-12-10

**Authors:** Michel B Araújo, Leandro P Moura, Roberto C Vieira Junior, Marcelo C Junior, Rodrigo A Dalia, Amanda C Sponton, Carla Ribeiro, Maria Alice R Mello

**Affiliations:** 1 Laboratory of Nutrition, Metabolism and Exercise, Department of Physical Education, Universidade Estadual Paulista (UNESP), 24ª avenue nº 1515, P.O. Box 199, Bela Vista, Rio Claro, São Paulo, Brazil; 2 Post-Graduation Program – Graduation in Bioscience of Faculty of Nutrition from Federal University of Mato Grosso, UFMT, Cuiabá, Mato Grosso, Brazil

**Keywords:** Creatine supplementation, Oxidative stress, Enzymes, Treadmill exercise

## Abstract

**Background:**

The objective of this study was to determine the effects of creatine supplementation on liver biomarkers of oxidative stress in exercise-trained rats.

**Methods:**

Forty 90-day-old adult male Wistar rats were assigned to four groups for the eight-week experiment. Control group (C) rats received a balanced control diet; creatine control group (CCr) rats received a balanced diet supplemented with 2% creatine; trained group (T) rats received a balanced diet and intense exercise training equivalent to the maximal lactate steady state phase; and supplemented-trained (TCr) rats were given a balanced diet supplemented with 2% creatine and subjected to intense exercise training equivalent to the maximal lactate steady state phase. At the end of the experimental period, concentrations of creatine, hydrogen peroxide (H_2_O_2_) and thiobarbituric acid reactive substances (TBARS) were measured as well as the enzyme activity of superoxide dismutase (SOD), glutathione peroxidase (GSH-GPx) and catalase (CAT). Liver tissue levels of reduced glutathione (GSH), oxidized glutathione (GSSG) and the GSH/GSSG ratio were also determined.

**Results:**

Hepatic creatine levels were highest in the CCr and TCr groups with increased concentration of H_2_O_2_ observed in the T and TCr animal groups. SOD activity was decreased in the TCr group. GSH-GPx activity was increased in the T and TCr groups while CAT was elevated in the CCr and TCr groups. GSH, GGS and the GSH/GSSG ratio did not differ between all animal subsets.

**Conclusions:**

Our results demonstrate that creatine supplementation acts in an additive manner to physical training to raise antioxidant enzymes in rat liver. However, because markers of liver oxidative stress were unchanged, this finding may also indicate that training-induced oxidative stress cannot be ameliorated by creatine supplementation.

## Background

Physical activity modifies the balance between oxidative stress and antioxidant defense mechanisms. For both athletes and fitness enthusiasts, the combination of regular physical activity and antioxidant supplementation may have important restorative effects on the body’s oxidation-reduction or redox balance.

Dietary supplementation with creatine (CrS) is popular in the sports and fitness industry, wherein CrS is believed to aid in the maintenance of high-energy phosphate reserves during exercise. While certain mechanisms of action involved in improved physical exercise performance with CrS have been established [1,2], recent research efforts have focused on other CrS benefits, specifically, the use of CrS in reducing the cellular oxidative stress associated with strenuous long-term exercise [3-5]. Creatine is an end-product of the metabolism of amino acids glycine and arginine, producing guanidinoacetate and participating in the urea cycle. Arginine also acts as a substrate in the nitric oxide synthase pathway and can stimulate the production of nitric oxide free radicals that modulate skeletal muscle and liver metabolism, contractility and glucose uptake [6-8].

Certain amino acids such as histidine, methionine and cysteine are particularly susceptible to oxidation by free radicals [9]. Sulfhydryl cysteine groups are known modulators of the redox state across many protein functions that also appear to protect protein sulfhydryl groups and to improve liver function [10].

The antioxidant effects of creatine may derive from different mechanisms of action such as the indirect mechanisms involved in cell membrane stabilization and improved cellular energy capacity [11] and from its direct antioxidant properties [5]. Recently, creatine’s potential to act directly to remove reactive oxygen species was investigated [12]. Lawler et al. [5] concluded that creatine has a significant role as a primary antioxidant. Using in vitro techniques, these authors found a dose-response relationship between creatine concentration and the ability to remove superoxide anions (O_2_•-) and peroxynitrite (OONO). As creatine has not shown significant antioxidant activity against hydrogen peroxide (H_2_O_2_), these findings also demonstrate creatine’s selective antioxidant capacity.

Sestili et al. [4] postulated a direct antioxidant role for creatine in cells exposed to various oxidative agents. These authors demonstrated that creatine in doses similar to those found in plasma after supplementation exerts cytoprotective antioxidant activity in three different cell lines against three different oxidative agents: H_2_O_2_, OONO^-^ and t-butyl hydroperoxide (tB-OOH), an organic peroxide widely used in a variety of oxidation processes. Furthermore, cytoprotection was observed independent of the anti-oxidative state of the cell, as evaluated by the antioxidant enzymes catalase and glutathione peroxidase, which suggests a direct interaction between creatine and oxidizing agents and/or free radicals.

In humans, creatine synthesis appears to occur mainly in the liver [13], an organ that requires vast amounts of generated energy to perform its various functions. The high metabolic rate of the liver (200 kcal/kg of tissue per day) is directly associated with the high flow of electrons in the mitochondrial respiratory chain [14]. However, some of these electrons are diverted to produce reactive oxygen species (ROS). Several authors have demonstrated that the liver undergoes increased oxidative stress following exercise [14,15].

Thus, we sought to investigate the effects of CrS on oxidative balance, injury and liver antioxidant defense mechanisms during exercise in a laboratory model. The aims of this study were to: 1) determine whether creatine supplementation increased liver creatine stores and 2) determine whether creatine supplementation improved markers of liver oxidative stress following exercise training.

## Methods

### Animals and treatment

Forty 90-day-old male Wistar rats were given free access to water and food. The animals were housed in collective polyethylene cages measuring 37.0 × 31.0 × 16.0 cm with 5 animals per cage, all under controlled conditions of temperature (22°C) and light/dark cycle (12 h/12 h). The experiment was submitted to and approved by the Animal Experimentation Ethics Committee at the University of Taubaté - UNITAU, São Paulo State, Brazil (register CEEA / UNITAU n° 018/08).

Exercise training was performed and creatine supplementation given over eight weeks with animals allocated into four groups of ten animals in each group: control group (C), sedentary rats that received a balanced control diet; creatine control group (CCr), sedentary rats that received a balanced diet supplemented with 2% creatine; trained group (T), rats that were subjected to a training protocol and received a balanced diet; and supplemented trained group (TCr), rats that were subjected to a training protocol and received a balanced diet supplemented with 2% creatine.

### Diet

The animals in the creatine-supplemented groups (CCr and TCr) received the balanced, isocaloric AIN-93 M diet [16] supplemented with 13% or 2% monohydrated creatine (All Chemistry, São Paulo, SP, Brazil) [17].

According to Hutman et al. [18] and Vandenbergue et al. [19] creatine supplementation must be provided in two phases, which aims to promote an overload state of this substrate. These phases were designated as a first peak phase and a subsequent maintenance phase. During the peak phase, rats received the 13% creatine diets for seven days followed by a maintenance phase for the remaining days of the experiment during which rats were fed a 2% creatine diet. We used the dosage of creatine based on dose for human but there was an adjustment for employment with the animals. The addition of 2% in diet creatine during the maintenance phase equals 20 g.kg-1 peak in the phase of 13% were used equivalent to 130 g.kg-1. Still, according to Altman and Dittmer [20], sets the speed rat metabolism is 5 times greater than the human being for this reason these present values of creatine supplementation. Thus, animals that received creatine-supplemented feed were supplemented seven days a week for eight weeks of the experiment.

The animals from groups C and T received the balanced isocaloric diet AIN-93 M [16] without addition of creatine. The detailed diet composition is provided in Table 1.

**Table 1:** Diets compositions

**Components**	AIN – 93M*	Addition of 2% creatine**	Addition of 13% creatine***
	**(g_kg–1)**		
		**(g_kg–1)**	**(g_kg–1)**
Creatine	0.0	20.0	130.0
Cornstarch	465.7	444.7	335.7
Casein (85% protein)	140.0	140.0	140.0
Dextrin	155.0	155.0	155.0
Sucrose	100.0	100	100
Soybean oil	40.0	40.0	40.0
Fiber	50.0	50.0	50.0
Mineral mix	35.0	35.0	35.0
Vitamin min	10.0	10.0	10.0
L-cystine	1.8	1.8	1.8
Choline bitartrate	2.5	2.5	2.5
Kcal/Kg	3.802,77	3.802,77	3.802,77

*American Institute of Nutrition (AIN-93M) [16].

**Creatine maintenance diet according to Demenice et al. [17].

***Creatine peak diet addapted from Demenice et al. [17] and according to Hultman et al. [18] and Vandenbergue et al. [19].

### Training protocol

To determine the Maximum Lactate Steady State (MLSS), series of exercises was performed, rats bearing rectangular loads ran for 25 minutes on a treadmill at different fixed speeds for each series and a 48-hour interval between series. Blood sample was obtained every five minutes for lactate measurement and were taken from a small incision at the end of the tail that was made prior to the beginning of exercise and was sufficient for all specimen collections. The blood lactate concentration representative of the MLSS was considered that obtained from the highest speed where there was no variation in blood lactate between 10 and 25 min of exercise was no greater than 1.0 mmol/L [10,20]. The blood lactate concentration was determined by an enzymatic method [21].

The average MLSS for all rats was 26 m/min. Thus, all rats were trained at this intensity for 40 minutes/day, five days/week for the duration of the experiment.

### Biochemical analysis

Animals were sacrificed after anesthesia with CO_2_ at the conclusion of the experiment in the fed state and 48 hours after the last “in vivo” evaluation. Blood, liver and gastrocnemius muscle were removed for creatine measurement as described by Clark [22]. As biomarkers of oxidative stress, H_2_O_2_ was determined as hydrogen peroxide (Amplex UltraRed Reagent® kit, Life Technologies Corporation, Grand Island, New York, USA) and thiobarbituric acid reactive substances (TBARS) [23] were also evaluated.

As indicators of the antioxidant system, enzymatic activity was analyzed for superoxide dismutase (SOD) (Cayman Chemical commercial kit, Ann Arbor, Michigan, USA), glutathione peroxidase (GSH-GPx) (Cayman Chemical commercial kit, Ann Arbor, Michigan, USA) and catalase (CAT) [24]. Glutathione, both reduced (GSH) and oxidized (GSSG), were analyzed according to the method of Hissin and Hilf [25].

### Statistical analysis

The normality of the data was confirmed by the Shapiro-Wilks test. The results are presented as the mean ± S.E. (standard error). Comparisons between groups were made through an analysis of variance (ANOVA *Two-Way*) and the Tukey HSD post-hoc test when necessary. A predetermined 5% significance level was used for all the analyses. The statistical program used was the STATISTICA®, version 7.0.

## Results

### Creatine concentration in the liver

**Figure 1:**
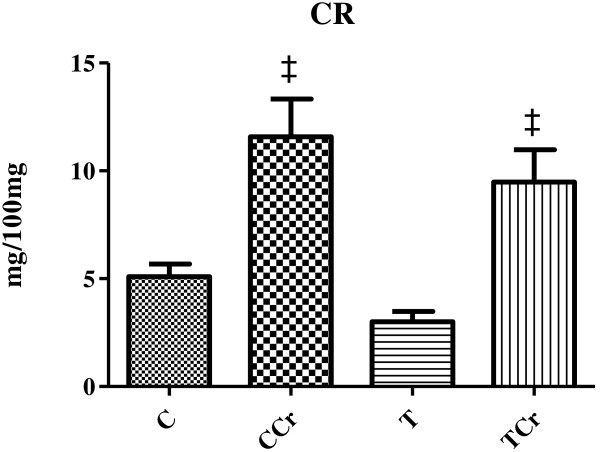
**Creatine concentration (CR) in the liver the animals at the end of the experiment.** The results are expressed as the mean + S.E. of 10 animals per group. TCr = Trained creatine; T = Trained; CCr = Control Creatine; C = Control not trained. ‡ different T/C.

Animals supplemented with creatine showed significant increase in hepatic creatine concentration when were compared to animals that received no supplementation (Figure 1).

### Concentration of hydrogen peroxide (H_2_O_2_) and thiobarbituric acid reactive substances (TBARS) in the liver

**Figure 2:**
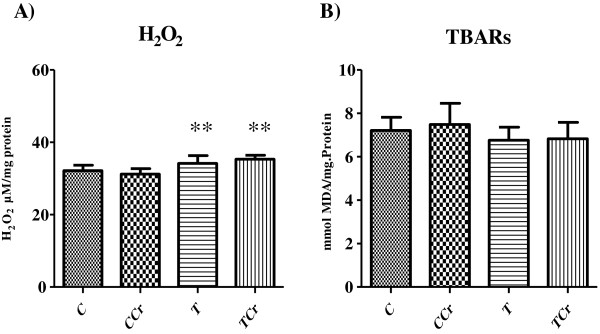
**Analysis of pro-oxidants. A)** Concentration of H_2_O_2_**B)** Concentration of TBARs. The results are expressed as the mean + S.E. of 10 animals per group. TCr = Trained Creatine; T = Trained; CCr = Control Creatine; C = Control not trained ** different C e CCr.

Liver H_2_O_2_ levels obtained at the end of the experiment were significantly increased in the exercise-trained groups T and TCr in relation to control groups C and CCr (Figure 2A).

The values for hepatic TBARS at the end of experiment did not differ between groups (Figure 2B).

### Activity of superoxide dismutase (SOD), glutathione peroxidase (GSH-GPx) and catalase (CAT) in the liver

**Figure 3:**
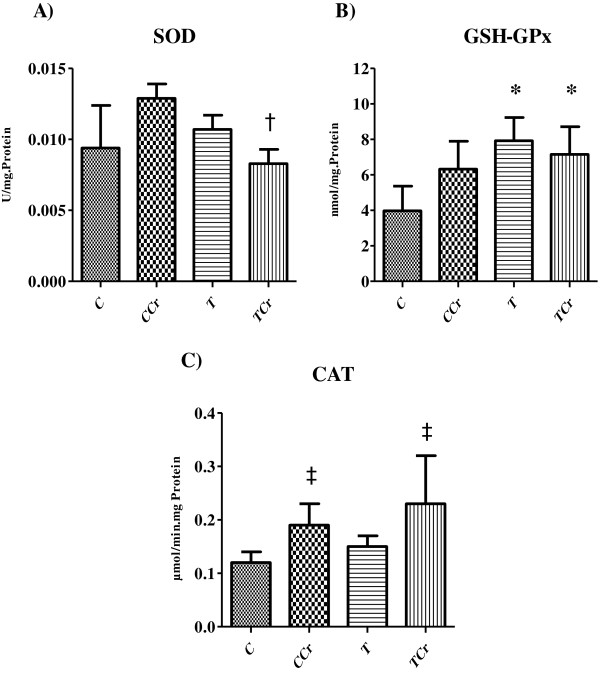
**Analysis of antioxidants. A)** Activity of SOD; **B)** GSH-GPx and **C)** CAT. The results are expressed as the mean + S.E. of 10 animals per group. TCr = Trained Creatine; T = Trained; CCr = Control Creatine; C = Control not trained. * different C; † different CCr; ‡ different T/C.

Hepatic SOD activity at the end of the experiment showed decreased activity in rats from the TCr group when they were compared with rats from CCr group (Figure 3A). Hepatic GSH-GPx activity at the end of the experiment was elevated in groups T and TCr compared with group C rats (Figure 3B). Values obtained for hepatic CAT activity at the end of the experiment showed no differences between groups (Figure 3C). There was an increase in the activity of CAT in rats from the CCr and TCr groups in relation to those in control groups C and T (Figure 3C).

### Concentration of reduced glutathione (GSH), oxidized glutathione (GSSG) and ratio between reduced glutathione and oxidized glutathione (GSH/GSSG) in liver

**Figure 4:**
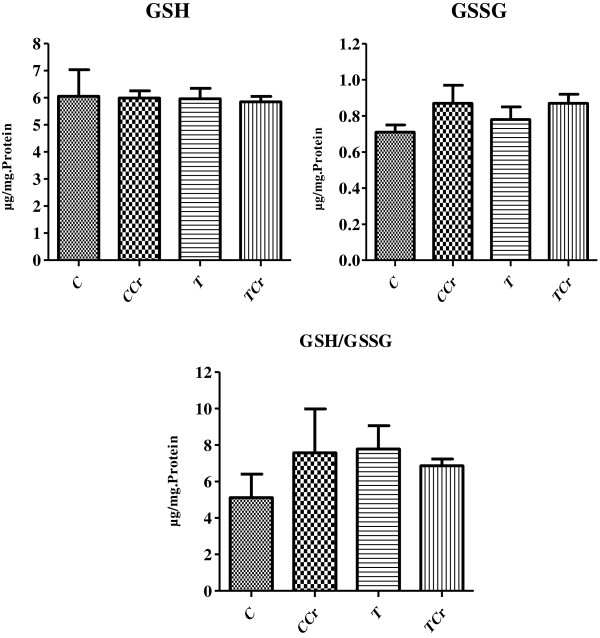
**Concentration of reduced glutathione, oxidized glutathione and ratio reduced glutathione/oxidized glutathione in the liver the animals at the end of the experiment.** The results are expressed as the mean + S.E. of 10 animals per group. TCr = Trained Creatine; T = Trained; CCr = Control Creatine; C = Control not trained.

Rat liver values for GSH, GSSG and GSH/GSSG ratio at the end of the experiment showed no differences between groups (Figure 4).

## Discussion

In recent years the use of creatine supplementation (CrS) whith antioxidant function has increased. Several studies have confirmed these effects and pointed to creatine as a new alternative in the prevention of oxidative stress in which creatine appears to play a crucial role in reducing the toxic effects of endogenous production of reactive oxygen species (ROS) [5,26-28].

The literature indicates that 2% CrS in animal feed is able to trigger a significant increase in phosphocreatine (PCr) and creatine levels in rat tissues [29,30]. Using this amount of creatine, McMillen et al. [30] observed a significant increase in the total creatine content of rat gastrocnemius muscle in two weeks of supplementation. In the present study, significant increase in the hepatic creatine concentrations were demonstrated in CCr and TCr rats compared to the non-supplemented control groups, which supports prior findings in the literature [30,31].

After confirming that dietary supplementation increased creatine concentration in rat liver, this study aimed to evaluate the possible antioxidant effects of CrS in vivo. The results demonstrate that creatine exerts indirect antioxidant activity in rat liver, i.e., creatine increased the activity of antioxidant enzymes GSH-GPx and CAT. However, CrS was not effective in normalizing the increased concentrations of H_2_O_2_ triggered by exercise. In addition, no significant differences were observed in the concentration of TBARS between groups.

H_2_O_2_ plays an important role in homeostasis. It participates in cellular induction of gene expression, among which are those genes responsible for antioxidant enzyme synthesis [32-34]. In the present study, we demonstrated that exercise-trained rats (T and TCr) had higher concentrations of H_2_O_2_ than sedentary rats (C and CCr). These data reinforce the observations of several authors that indicate that creatine appears to exert selective antioxidant effects [26,27]. Lawler et al. [5] have shown that creatine was able to remove O_2_•- and OONO^-^ but had no effect on H_2_O_2_. Thus, the direct antioxidant actions of creatine appear to be limited to certain types of free radicals or reactive oxygen species. Sestili et al. [4] have found that creatine was not able to significantly counteract the concentrations of H_2_O_2_ and the compound tB-OOH that is derived from •OH and RO• radicals.

With regard to levels of TBARS, our results are consistent with previous findings [35] that showed no change in hepatic TBARS levels in treadmill exercise-trained rats. Taken in aggregate, these results for pro-oxidant markers underscore the findings of Sjodin et al. [36] and Souza et al. [37], that is, predominantly aerobic exercise causes increased oxygen flow in the mitochondria and approximately five percent of this oxygen is not completely reduced, thereby forming ROS.

As H_2_O_2_ levels rise, homeostasis requires increased production of antioxidant enzymes such as SOD, GSH-GPx and CAT to maintain the balance between oxidant production and the antioxidant system [8,38,39]. Our study results for SOD demonstrate decreased enzymatic activity in trained animals (T and TCR) when they were compared to group C rats. SOD is important in the metabolism of O2•- that results in the formation of H_2_O_2_[34,40,41]. Thus, while SOD is an important combatant against oxidative stress, it also accelerates the formation of hydrogen peroxide, as occurs during physical exercise. In this situation, it has been suggested that reduced SOD activity is mainly explained by the inhibitory effect of increased H_2_O_2_ production [42].

In this study, a hypothesis may explain the decrease in SOD activity in response to CrS. Creatine may exert a sparing effect, i.e., creatine may act to neutralize ROS, resulting in down-regulation of the antioxidant system and specifically, the action of SOD. This hypothesis is based on research of antioxidant supplementation use that demonstrated inhibition of SOD, GSH-GPx and CAT activity [43,44]. However, a notable finding from these studies was that unlike SOD, the activity of GSH-GPx and CAT were increased in trained animals and CrS. Both GSH-GPx and CAT enzymes are present in most aerobic organisms and are responsible for conversion of intracellular H_2_O_2_ to water and oxygen [34,40].

Our study demonstrated increase in GSH-GPx levels in exercised-trained rat groups T and TCr compared to control group animals. This finding may be explained by the fact that regular physical training activates transcription factors such as NF-κB and Nrf2, which are responsible for triggering various genes, including mitochondrial GSH-GPx [45,46]. Moreover, the effect of training on the activity and expression of CAT is inconsistent and controversial [47]. However, increased activity of this enzyme has been observed in rat liver [48], mice liver [49] and trained rat heart [50].

The most striking finding we encountered was the increased CAT activity that occurred only in animals given CrS. This result appears to support an additive role for creatine on the actions of antioxidant enzymes. Physical training, as demonstrated by Halliwell and Gutteridge [51], activates transcription factors such as AMPK, which activate CAT mRNA, thereby stimulating protein synthesis and possibly increasing CAT activity. The ability of CrS to also exert this effect remains controversial. According to Sestile et al. [4], creatine has neutralizing effects on ROS production that do not interfere on the action of antioxidant enzymes. However, the increase in CAT activity observed in this study is attributed to the formation of H_2_O_2_ by SOD. According to Halliwel and Gutteridge [51], the chemical interaction of H_2_O_2_ at the catalase active site involves the transfer of a hydrogen ion between the two oxygen atoms, causing a heterolytic cleavage with water and oxygen end products. The findings in our study of increased H_2_O_2_ levels in trained and supplemented animals combined with the neutralizing action of creatine on this ROS may explain the reduced oxidative damage seen with increased CAT activity.

In contrast, the amounts of GSH and GSSG as well as the ratio between GSH/GSSG did not differ between the study groups. GSH has a central role in the biotransformation and elimination of xenobiotics, and protects cells against oxidative stress [52]. To maintain the protective activity of glutathione as expressed by the reduction of oxidizing species and consequent oxidation of GSH to GSSG, GSH must be regenerated through the catalytic cycle [52].

In summary, our study results demonstrate that creatine supplementation acts in an additive manner to physical training to increase antioxidant enzymes in rat liver. More studies are needed to expand our knowledge of the antioxidant effects of creatine and to investigate creatine’s little-known effects on other body tissues.

## Competing interests

The authors declare that they have no competing interests.

## Authors’ contributions

MBA (corresponding author) was responsible for the study design, execution of biochemical analysis, statistical analysis and writing of the manuscript. LPM held the writing of the manuscript. RCVJ, MCJ, RAD, ACS, CR and MARM participated in the realization of biochemical analysis. All authors read and approved the final manuscript.
